# Regulation of epidermal cell fate in Arabidopsis roots: the importance of multiple feedback loops

**DOI:** 10.3389/fpls.2014.00047

**Published:** 2014-02-17

**Authors:** John Schiefelbein, Ling Huang, Xiaohua Zheng

**Affiliations:** Department of Molecular, Cellular, and Developmental Biology, University of MichiganAnn Arbor, MI, USA

**Keywords:** root hairs, transcription factors, pattern formation, feedback loops, *Arabidopsis thaliana*

## Abstract

The specification of distinct cell types in multicellular organisms is accomplished via establishment of differential gene expression. A major question is the nature of the mechanisms that establish this differential expression in time and space. In plants, the formation of the hair and non-hair cell types in the root epidermis has been used as a model to understand regulation of cell specification. Recent findings show surprising complexity in the number and the types of regulatory interactions between the multiple transcription factor genes/proteins influencing root epidermis cell fate. Here, we describe this regulatory network and the importance of the multiple feedback loops for its establishment and maintenance.

## Epidermal cell patterning in the arabidopsis root

**Figure 1:**
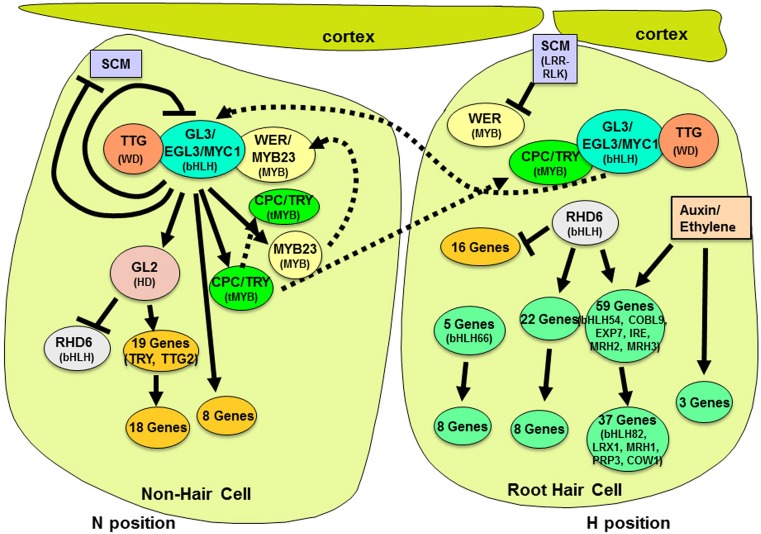
**Molecular genetic regulation of cell fate specification in the Arabidopsis root epidermis**. Root-hair specific gene expression occurs in differentiating epidermal cells located in a cleft between adjacent cortical cells (right-most cell). Gene regulatory activity is indicated by solid lines with arrows (positive transcriptional effect) or bars (negative transcriptional effect). Dotted lines represent the movement of proteins within or between cells. The downstream genes shown here are taken from Bruex et al. (2012) and defined as hair or non-hair genes based on their accumulation in the root epidermis in either *cpc try* (non-hair) mutants vs. *ttg1*, *wer myb23*, or *gl3 egl3* (hairy) mutants.

The specification of root hair cells and non-hair cells in the Arabidopsis root is a well-studied model for understanding cell fate decisions in plants (Schiefelbein et al., 2009; Tominaga-Wada et al., 2011; Grebe, 2012). Newly formed epidermal cells located outside the cleft separating two adjacent underlying cortical cells (the “H” cell position) differentiate into root-hair cells, whereas epidermal cells not located over the cleft (the “N” cell position) develop into non-hair cells, due to differential cell-type-specific gene expression (Figure 1) (Cormack, 1962; Berger et al., 1998; Bruex et al., 2012). Genetic and molecular studies over the past 20 years have now provided a fairly clear picture of the transcriptional regulators responsible for establishing this differential cell-type gene expression. What has been surprising is the large number of regulatory mechanisms and interactions by these transcription factors in the process of root epidermal cell specification. In this mini-review, we describe the basic transcription factor components and then we outline the many categories of regulatory mechanisms and their roles in establishing the epidermal cell fates.

## The basic components of the network

At its core, cell fate in the root epidermis is dependent on the relative abundance of a transcription factor complex consisting of three types of proteins: the Myb-domain protein WEREWOLF (WER) (Lee and Schiefelbein, 1999), two redundantly acting bHLHs GLABRA3 (GL3) and ENHANCER OF GLABRA3 (EGL3) (Payne et al., 2000; Bernhardt et al., 2003, 2005; Simon et al., 2013), and the WD-repeat TRANSPARENT TESTA GLABRA (TTG1) (Galway et al., 1994). Differentiating epidermal cells that generate a significant amount of this WER-bHLH-TTG complex express the downstream HD-ZIP gene *GLABRA2* (*GL2*), which represses transcription of the hair-cell promoting bHLH *ROOT HAIR DEFECTIVE 6* (*RHD6*) (Masucci and Schiefelbein, 1994; Menand et al., 2007), leading to the expression of dozens of non-hair-cell-specific genes and the non-hair cell fate (Figure 1) (Masucci et al., 1996; Bruex et al., 2012). Differentiating cells that do not accumulate a significant amount of the WER-bHLH-TTG complex are able to express *RHD6*, and as a result, initiate transcription of hundreds of root-hair-cell-specific genes (Cvrckova et al., 2010; Bruex et al., 2012). Given the key role of the WER-bHLH-TTG transcriptional complex for the cell fate decision, there has been great interest in defining and understanding the mechanisms that regulate its accumulation in the two cell types. Recent research has uncovered an array of intra- and intercellular mechanisms responsible for controlling the abundance of this key complex.

## Regulatory mechanisms in the network

### Lateral inhibition

The activity of the WER-bHLH-TTG complex is inhibited by a set of small, one-repeat Myb proteins, which includes CAPRICE (CPC), TRIPTYCHON (TRY), and ENHANCER OF TRY AND CPC1 (ETC1) (Wada et al., 1997; Schellmann et al., 2002; Kirik et al., 2004; Simon et al., 2007). These proteins are able to bind to the GL3/EGL3 bHLHs, competitively inhibiting WER binding and generating a non-functional complex (Lee and Schiefelbein, 2002). Accordingly, these proteins accumulate in the H-position cells, where they promote the hair cell fate (Kurata et al., 2005; Kang et al., 2013). Unexpectedly, the transcription of these one-repeat Mybs was found to occur predominately in the N cells, due to positive regulation by the WER-bHLH-TTG complex itself, and the proteins appear to move through plasmodesmata to accumulate in the H cells (Kurata et al., 2005; Kang et al., 2013). The ability of cells adopting the non-hair fate (accumulating WER-bHLH-TTG) to generate diffusible molecules (CPC/TRY/ETC1) that prevent adjacent cells from adopting the same fate (via inhibition of the WER-bHLH-TTG action) effectively represents a kind of lateral inhibition mechanism, a general strategy widely employed by multicellular organisms to establish distinct cell identities in an initially equivalent field of cells (Meinhardt and Gierer, 2000). What is unusual about the lateral inhibition used here is its direct nature; the molecule produced by the inhibiting cell is used as both the signal and the inhibitor of the recipient cell.

### Feedback at multiple developmental times

Although the *CPC*, *TRY*, and *ETC1* genes are all positively regulated by the WER-bHLH-TTG complex, the effect on *TRY* is indirect because it is downstream of the N-cell regulator GL2 (Figure 1) (Simon et al., 2007). This means that TRY production will be developmentally delayed, relative to CPC and ETC1. Since the CPC/ETC1 and TRY proteins are members of different subtypes and appear to vary in their properties (Pesch and Hulskamp, 2011), this regulatory organization may generate different ratios of subtypes during epidermis development important for pattern establishment.

### Positive feedback

The MYB23 protein is the Arabidopsis MYB most closely related to WER, and MYB23 is capable of substituting for WER in root hair development (Kang et al., 2009). Further, the *MYB23* gene is under the positive transcriptional regulatory control of the WER-bHLH-TTG complex. Taken together, these data indicate that the *MYB23* gene participates in a positive feedback loop in the N cells (Figure 1), which apparently is used to maintain relatively high levels of the complex, due to MYB23's presumed participation in the complex (Kang et al., 2009). The identification of a positive feedback loop affecting the WER-bHLH-TTG complex was satisfying, because theoretical models of lateral inhibition in pattern formation typically require such self-promoting loops to create stable peaks of activator accumulation even in the presence of high inhibitor levels (Meinhardt and Gierer, 1974, 2000). Accordingly, in the root epidermal system, mutants lacking *MYB23* function are less able to adopt appropriate cell fate decisions (Kang et al., 2009).

### Mutual reinforcing loops

The *GL3* and *EGL3* bHLH genes were found to be preferentially transcribed, and to have their transcripts preferentially accumulate, in the H cells rather than the N cells of the developing root epidermis, due to negative transcriptional regulation of these genes by the WER-bHLH-TTG complex (Bernhardt et al., 2005). This was an unexpected finding, since the GL3 and EGL3 proteins are required for the proper function and differentiation of the N cells. Based on the use of GFP-tagged proteins, the GL3/EGL3 gene products were found to preferentially accumulate in the N cells, implying movement of these proteins from H to N cells (Figure 1). The opposite movement of the hair-promoting CPC/TRY/ETC1 (N to H cells) vs. the non-hair-promoting GL3/EGL3 (H to N cells) has been proposed to represent an intercellular mutual reinforcing loop that exists to provide robustness to the patterning system (Figure 1) and this view has received support from theoretical modeling studies (Savage et al., 2008; Benitez and Alvarez-Buylla, 2010).

### Molecular trapping

The observed preferential accumulation of CPC (and presumably TRY and ETC1) in the H cells is believed to be necessary for robust pattern formation, though the mechanism responsible for causing these mobile factors to accumulate in H cells has long been a mystery. A possible explanation has recently been provided by the finding that this accumulation is EGL3 dependent (Kang et al., 2013). A CPC-GFP fusion protein, expressed under control of the *CPC* or *SHORTROOT* (*SHR*) promoter, lacked preferential H-cell accumulation in the *gl3 egl3* mutant and exhibited reduced movement in GL3/EGL3 overexpression lines (Kang et al., 2013). One possibility is that EGL3 serves to “trap” the CPC inside the H cells by relatively strong binding (Kang et al., 2013). This is at least superficially similar to the proposed nuclear trapping of TTG1 by GL3 in the trichome specification system (Balkunde et al., 2011).

### Feedback on positional signaling

The position-dependent pattern of hair and non-hair cells is dependent on signaling through the SCRAMBLED (SCM) receptor-like kinase (Kwak and Schiefelbein, 2007). SCM action leads to reduced *WER* transcription, and since this appears to preferentially occur in the H cell position, it explains how SCM signaling helps generate the cell-type pattern. Interestingly, the preferential SCM action in the H cells is likely due to differential accumulation of SCM. This differential SCM accumulation is achieved by a negative feedback loop between the WER-bLHLH-TTG complex and the *SCM* gene expression, ensuring reduced SCM signaling in the N position (Kwak and Schiefelbein, 2008). It is proposed that this mechanism helps to “lock in” the cell fate decision, by amplifying the differential SCM signaling ability of the two cell types.

### Regulation by hormones

Root hair development is affected by several plant hormones, most commonly reported for auxin and ethylene (Masucci and Schiefelbein, 1996; Pitts et al., 1998). In general, the hormones appear to promote root hair formation, because addition of exogenous hormone typically leads to increased hair length or number whereas inhibition of hormone production/action tends to reduce hair length or number. Indeed, transcriptome assays show that a majority of the downstream root hair genes, but not the non-hair genes, are responsive to addition of auxin (IAA) or ethylene (ACC) (Bruex et al., 2012). Thus, the accumulation and activity of hormone pathway elements provide the opportunity for regulation of root-hair pattern at a relatively late stage, perhaps allowing for environmental influence of root hair formation.

### Regulation by histone modification

The expression of the patterning genes and the arrangement of the root cell types are influenced by histone acetylation. Treatment of roots with trichostatin A (histone deacetylase inhibitor) or mutations in the histone deacetylase gene *HDA18* cause N-position cells to become root hair cells (Xu et al., 2005). Because the HDA18 protein does not directly bind to the patterning gene promoters (Liu et al., 2013), there may be an intermediate set of histone-regulated genes responsible for this level of control.

## Thoughts on the complexity of the network

In this minireview, we have highlighted the multitude of regulatory mechanisms that are employed to control the relative abundance of the critical transcription factors in epidermal cell specification. Considering these many components and interactions (Figure 1), it is appropriate to wonder why this system has evolved such complexity to control a seemingly simple case of cell fate specification. One possible explanation is that the complex regulatory interactions reflect a requirement for robustness; to ensure that once a cell fate decision is made, that this decision is fully adopted and is not allowed to be altered at any step (Barkai and Leibler, 1997; Benitez and Alvarez-Buylla, 2010). Another possibility for the existence of multiple regulatory mechanisms may be that they provide opportunities for the modification/adjustment of the cell fate decision at many points in the process, perhaps enabling it to respond to the many known internal and external factors that influence root hair development (Forde and Lorenzo, 2001; Datta et al., 2011). Future studies on the control of root epidermal cell fate in Arabidopsis and other species will likely yield additional insight into the importance of the many components and interactions in this complex regulatory network.

### Conflict of interest statement

The authors declare that the research was conducted in the absence of any commercial or financial relationships that could be construed as a potential conflict of interest.
